# Automating Motivational Interviewing Coding in Adolescent Substance Use Prevention: Human-AI Agreement Study

**DOI:** 10.2196/95964

**Published:** 2026-08-25

**Authors:** Francisco Cardozo, Eric C Brown, Juliana Mejía-Trujillo, Raymond Balise, Catalina Cañizares, Augusto Pérez-Gómez, Sara M St George, Vilma Gabbay

**Affiliations:** 1 Miller School of Medicine, University of Miami, 1252 Memorial Dr, Coral Gables, Miami, FL, 33146, United States, 1 305-284-2211; 2 Corporación Nuevos Rumbos, Bogotá, Colombia; 3 Applied Psychology, New York University, New York, NY, United States; 4 Nathan Kline Institute for Psychiatric Research, Orangeburg, NY, United States

**Keywords:** motivational interviewing, adherence, implementation fidelity, large language models, artificial intelligence, preventive interventions, intercoder agreement

## Abstract

**Background:**

Motivational interviewing (MI) is widely used in preventive interventions, yet coding MI techniques and monitoring intervention adherence remain resource-intensive due to the reliance on manual transcription and expert review. Large language models (LLMs) offer a promising approach to automate these tasks, but their agreement with human coders in the context of prevention interventions has not been established.

**Objective:**

This study evaluated the agreement between an AI-based coder (OpenAI’s GPT 4.1) and trained human coders on two tasks: (1) identification of MI techniques (eg, open questions, affirmations, giving information) at the facilitator-message level and (2) completing a 21-item checklist of implementation adherence for a brief MI-based preventive intervention for adolescent substance use.

**Methods:**

Two certified MI facilitators independently coded 72 facilitator messages from 2 standardized Spanish-language Brief Intervention Based on Motivational Interviewing program (Intervención Breve Basada en Entrevista Motivacional [IBEM]) practice sessions with chatbot-simulated adolescent responses. The facilitators classified MI techniques using the OARS (open questions, affirmations, reflections, and summaries) framework and completed a 21-item implementation-adherence checklist. An AI-based coder (OpenAI’s GPT-4.1, accessed through the API) classified the same facilitator messages and checklist items using a structured prompt derived from the MI coding manual. MI techniques were compared at the facilitator-message level and implementation adherence at the session level. Intercoder agreement in use of MI techniques and implementation adherence was assessed using Cohen κ, Fleiss κ, and Cochran *Q* tests.

**Results:**

For use of MI techniques, the AI coder demonstrated moderate-to-substantial agreement with human coders across most techniques, including open questions (κ=0.66-0.69), affirmations (κ=0.66-0.77), and giving information (κ=0.91). No statistically significant differences in percentages of MI technique use were observed among the 3 coders, although agreement was the weakest for higher-inference categories such as complex reflections (κ=0.00). For MI implementation adherence, overall agreement was moderate (Fleiss κ=0.487), and pairwise agreement between the AI coder and 1 human coder was substantial (κ=0.67), exceeding the agreement observed between the 2 human coders (κ=0.53).

**Conclusions:**

These findings provide support for the feasibility of using LLMs to recognize MI techniques and assess implementation adherence. The results support a human-AI collaborative model in which the AI coder “precodes” facilitator messages and flags sessions for expert review, while human coders retain responsibility for higher-inference judgments and shift their effort from routine coding toward contextual review and coaching feedback. Because the analyses are based on only 2 sessions, these results should be interpreted as early-stage, proof-of-concept evidence rather than a basis for large-scale deployment. Future research should compare different LLMs and evaluate whether AI-assisted coding improves the scalability of routine implementation monitoring.

## Introduction

Motivational interviewing (MI) is a client-centered therapeutic approach designed to strengthen an individual’s motivation and commitment to behavior change [1]. In prevention programs, MI is often used to encourage positive health behaviors and reduce risk factors before problems escalate. A growing body of evidence supports MI’s ability to enhance program engagement [2], promote adherence [3], and sustain healthier behaviors across diverse populations [4].

To implement MI effectively, facilitators train communication competencies such as reflective listening, empathetic communication, and the ability to guide individuals through ambivalence [5]. These competencies are expressed in conversation through the use of open-ended questions, affirmations, reflective listening, and summaries, commonly referred to by the acronym OARS [1]. OARS are fundamental to MI because they help participants articulate their experiences, recognize their strengths, feel heard, and engage in purposeful reflection, ultimately fostering motivation and promoting healthier behavioral choices. However, developing proficiency in these skills requires extensive practice through realistic conversational scenarios, and maintaining fidelity over time remains a persistent challenge in real-world settings [6]. As a result, there is a growing need for scalable training and feedback systems that can support MI facilitators in refining and sustaining their delivery skills beyond initial certification.

Several methods have been developed to evaluate how facilitators can effectively deliver MI competencies. Two widely used approaches are the Motivational Interviewing Skill Code (MISC) [7] and the Motivational Interviewing Treatment Integrity (MITI) framework [8]. The MISC is a widely used coding system that classifies every facilitator and participant interaction within a session, allowing the analysis of conversation elements such as change talk and sustain talk [7]. The MITI framework was developed as a more practical fidelity tool, focusing on a smaller set of global competencies (eg, empathy, MI spirit) and behaviors (eg, reflections, questions, affirmations). These methods provide a structured way to monitor facilitator proficiency and offer targeted feedback in training and supervision contexts. A key limitation of these methods, however, is their reliance on time-consuming processes such as verbatim transcription and manual coding of facilitator-participant interactions. This dependence significantly limits their scalability and feasibility in routine training and implementation settings. Consequently, the use of these methods to evaluate facilitators’ implementation of MI techniques is often impractical.

Prior efforts to automate MI fidelity coding have applied a range of natural language processing (NLP) and text classification methods to this problem, including rule-based systems, bag-of-words models, recurrent neural networks [9], and transformer-based classifiers trained on annotated MI corpora [10,11]. These approaches have shown that automated coding of facilitator utterances is feasible and can approximate human-level reliability for selected MI techniques. However, they typically require large, labeled training datasets, domain-specific model fine-tuning, and substantial computational infrastructure, resources that are rarely available in real-world settings. Moreover, most of this work has been conducted on English-language clinical therapy sessions with adult populations, limiting its applicability to other intervention contexts.

Recent advances in generative AI, specifically large language models (LLMs) [12], offer a qualitatively different approach [13]. Unlike earlier NLP methods, LLMs can perform text classification tasks through in-context learning, that is, by following natural-language instructions in a prompt [14], without requiring labeled training data or model fine-tuning [13]. This capability dramatically lowers the technical barrier to entry and makes automated coding accessible to research teams that lack a specialized machine learning infrastructure. Early evidence suggests that LLMs can achieve competitive performance on psychotherapy coding tasks, including the detection of therapeutic techniques in session transcripts [15,16]. However, evidence remains limited on whether LLMs can identify MI techniques in non-English standardized practice sessions for preventive interventions designed for adolescents in school-based settings.

The present study addresses this gap by examining the feasibility of using an LLM to assess MI implementation within the context of a brief substance use preventive intervention for adolescents delivered in Spanish. Using transcripts from Spanish-language facilitator practice sessions for a school-based preventive intervention, in which a chatbot simulated adolescent responses, we evaluated whether an AI-based coder can reliably detect the use of MI techniques (specifically OARS) by comparing its recognition of MI techniques against those of trained human coders. Conceptually, these 2 coding tasks map onto distinct dimensions of implementation fidelity as described in the framework of Carroll et al [17]: quality of delivery, operationalized as the appropriate use of MI techniques at the facilitator-message level, and adherence, operationalized through a 21-item checklist capturing the prescribed stages of a brief MI-based intervention. By examining both dimensions, the current study provides a more comprehensive test of the feasibility of AI-assisted fidelity measurement than studies targeting a single dimension. We examined (1) intercoder agreement between human coders and an AI-based coder and (2) whether the AI coder differed systematically from human coders in its ability to recognize specific MI techniques and implementation adherence. We hypothesized that the AI-based coder would demonstrate moderate-to-substantial agreement with human coders across (1) commonly used MI techniques and (2) indicators of MI implementation adherence. We further hypothesized that the AI coder would not exhibit systematic bias relative to human coders.

## Methods

### Study Design

Facilitator messages from session transcripts were coded independently by trained human coders and an AI-based coder using a common MI coding frame. Agreement was examined at two levels: (1) at the level of facilitator messages for the identification of specific MI techniques and (2) at the session level, for assessing implementation adherence across intervention stages. Reporting follows the GRRAS (Guidelines for Reporting Reliability and Agreement Studies) for the intercoder agreement components [18] and is informed by the TRIPOD-LLM (Transparent Reporting of a Multivariable Prediction Model for Individual Prognosis or Diagnosis–Large Language Models) reporting guidance for studies that develop or evaluate LLMs [19]; completed checklists for both guidelines are provided Checklists 1 and 2.

### Intervention Context

The Brief Intervention Based on Motivational Interviewing program (*Intervención Breve Basada en Entrevista Motivacional* [IBEM]) served as the exemplar intervention for this study. IBEM is a brief, school-based preventive intervention, developed in Colombia, and grounded in the principles of MI. Its goal is to support adolescents in reflecting on their motivations and behaviors related to substance use, with the aim of promoting healthier decision-making and reducing risk-related behaviors. The intervention consists of a single core session followed by 2 follow-up meetings. The initial session, lasting approximately 15 to 20 minutes, centers on a personalized MI-guided conversation about alcohol and other substance use. Facilitators conduct a brief assessment; provide individualized feedback; and guide adolescents in exploring their motivations, perceived risks, and readiness for change. Through this dialogue, adolescents identify self-directed goals and develop strategies to reduce or prevent substance use. The 2 follow-up sessions are conducted at 3 and 6 months and focus on reviewing progress toward previously established goals, discussing achievements, and identifying barriers and facilitators that may influence behavior change. IBEM has been developed in Colombia and was implemented also in Mexico and Brazil. Evaluations of IBEM in Colombia have shown the intervention to be efficacious in reducing adolescent alcohol use and its associated risks [20,21]. In 2021, IBEM received the National Award for Best Practices in Prevention, granted by the Colombian Ministry of Health and Social Protection in recognition of its contribution to prevention practice.

### Participants

Two expert human coders were recruited in consultation with IBEM program developers [22] and were invited to participate in the study based on their prior certification in the intervention and demonstrated experience implementing it in school-based settings. Both coders were Colombian professionals and native Spanish speakers, reflecting the linguistic and cultural context in which IBEM was originally developed and delivered. Each coder held a professional degree in psychology and postgraduate-level training and completed formal instruction in MI. IBEM certification required a 32-hour training in adolescent substance use (ie, alcohol, tobacco, and other illicit drug use), MI principles and techniques, role-playing, and implementation evaluation. Both coders possessed over 5 years of experience delivering IBEM in real-world implementation contexts.

### IBEM Transcripts

Two transcripts were selected from a pool of 10 IBEM sessions conducted in Spanish by certified implementers, originally collected as part of a previous research project focused on developing an AI chatbot to simulate adolescent responses during IBEM delivery. IBEM sessions, therefore, were not routine clinical encounters; certified expert facilitators delivered complete IBEM sessions while interacting with a simulated adolescent, which allowed facilitators to exercise the full MI protocol under controlled conditions. The selection of IBEM sessions was based on three considerations: (1) expert review indicated that the sessions reflected typical IBEM delivery rather than either flawless delivery or substantial omission of protocol components, (2) sessions were of average length relative to the pool of sessions, and (3) coders were not assigned to transcripts from sessions they had personally facilitated. A facilitator message was defined as a single, uninterrupted facilitator turn in the transcript; when a turn contained several sentences, it was treated as 1 message and could receive multiple technique codes. The selected transcripts yielded 72 facilitator messages (32 messages from session 1 and 40 messages from session 2).

### Coding Framework

#### MI Techniques

The coding scheme was adapted from the MISC and the MITI frameworks and organized around the OARS. Consistent with these systems, facilitator behaviors were operationalized as discrete, message-level codes rather than global session ratings; higher-order MITI global dimensions (eg, empathy, MI spirit) were intentionally beyond the scope of this feasibility study. Each facilitator message was classified, independently by each coder, into one of the following categories: (1) reflections, (2) questioning, (3) confrontational, (4) affirmative, (5) giving information, and (6) not categorized. *Reflections* were coded when the facilitator repeated or paraphrased adolescent statements, with *simple reflections* mirroring content literally and *complex reflections* capturing underlying meaning or emotion. *Questioning* was distinguished as *open* (inviting elaboration) or *closed* (eliciting brief, yes or no responses). *Confrontational* included direct disagreement or corrective statements. *Affirmative* highlighted participant strengths or efforts. *Giving information* involved offering factual, neutral content related to alcohol, tobacco, or other illicit drug use. *Not categorized* was used for messages such as greetings or farewells that did not fall into the other categories. Coders were allowed to assign more than one technique to the same message. A provisional “unclear” category was initially available for messages whose function was ambiguous but was not endorsed by any coder during piloting and was dropped from the final scheme; residual ambiguous messages were coded as not categorized.

#### Implementation Adherence

Coders documented the *presence* (coded 1) or *absence* (coded 0) of IBEM intervention steps using a 21-item intervention adherence checklist that covered the 6 stages of the IBEM implementation protocol. In stage 1, Presentation and Contextualization assessed whether the facilitator introduced themselves, explained the program’s objectives, clarified voluntariness and confidentiality, and collected demographic data. Stage 2, Risk Assessment, captured whether facilitators reviewed student responses to the IBEM brief assessment surveys, explored recent behavior related to alcohol use in greater detail, communicated the risk level, and postponed notification of risk level until the end of the session when risk was severe. Stage 3, Evocation and Motivators, evaluated whether facilitators explored the student’s knowledge about alcohol, tobacco, and drugs, elicited personal motivators (eg, favorite activities), and linked these motivators to information about risks and consequences. Stage 4, Importance and Confidence, assessed whether the facilitator explained the scales, inquired about the reasons for scores, and explored the student’s confidence in their ability to change or maintain behaviors. Stage 5, Goal setting and Strategies, captured whether a concrete action plan and start date were established, and whether goals and strategies were generated by the student rather than the facilitator. Stage 6, Summary and Closure, evaluated whether the facilitator summarized key points of the session including risk level, motivators, effects, goals, and strategies, and provided information about the timing of the next session. Each of the 21 items was scored dichotomously at the session level, indicating whether the corresponding protocol component was *present* (coded 1) or *absent* (coded 0) in the session. The implementation-adherence score for a session was therefore the number of endorsed items out of 21, and agreement analyses were conducted across the 42 item-by-session ratings (21 items × 2 sessions) for each coder.

#### AI-Based Coding

An AI-based coder was developed using OpenAI’s GPT 4.1 model [23], accessed through the API. Facilitator messages were submitted to GPT 4.1 along with a structured prompt derived from the coding manuals. The prompt was designed to replicate the decision rules used by the human coders and included (1) definitions of each MI technique category (eg, reflections, questions, affirmations), (2) explicit instructions for distinguishing between similar categories (eg, open vs closed questions, simple vs complex reflections), and (3) guidance on how to handle ambiguous or irrelevant facilitator statements (eg, greetings or logistical remarks not otherwise categorized). The prompt also emphasized that multiple MI technique categories could be flagged simultaneously. To increase consistency in the AI responses, we instructed the model to return its classifications in a structured data format known as JSON. JSON is a simple way of representing information as a list of labels and values that computers can read. For each message, the model produced a JSON entry indicating whether each message belonged to an IBEM implementation protocol step (*TRUE* or *FALSE*) and included a short explanation for its decision. Classification outputs were then checked using the *Pydantic framework* [24], which validated whether the format of the AI’s responses followed the expected JSON structure. In practice, this meant verifying that every AI-generated response included the required pieces of information (ie, TRUE/FALSE decisions and the short explanation). The pipeline was implemented in *Python 3.12* [25], *Pandas* for data management [26], *PyArrow* for parquet handling [27], *Pydantic* for schema validation, and the official *OpenAI* module for API access [28]. In brief, JSON provided a consistent, machine-readable structure for each classification, pairing every category decision with a short textual rationale, and Pydantic enforced this schema so that any malformed or incomplete model output (eg, a missing decision field or an invalid category value) was automatically flagged and corrected before the AI codes were merged with the human-coded data set for analysis. This step was intended to improve the reproducibility and auditability of the automated coding rather than to alter the message classifications.

### Procedures

Human coders completed a structured virtual training based on the MI coding manual prior to transcript review. Coding involved 2 distinct tasks. First, coders evaluated MI techniques at the facilitator-message level, assigning codes to each individual facilitator message. Second, coders assessed intervention adherence at the item by session level using the 21-item checklist of required stages of IBEM implementation. After independently coding the first transcript, each human coder met individually with the research team to clarify ambiguities in the coding process. Minor refinements to category definitions were incorporated before the coding of the second transcript began. Coders were blinded to one another’s ratings and the AI-generated codes throughout the process. AI codes were generated in parallel and merged with the human-coded dataset for comparative analyses.

### Statistical Analysis

Intercoder agreement for MI techniques was assessed using Cohen κ [29], calculated separately for each technique category and pair of coders (coder 1 vs coder 2, coder 1 vs AI coder, and coder 2 vs AI coder). To examine whether coders differed systematically in their likelihood of identifying techniques for the same messages, Cochran *Q* tests were conducted for each technique across all 3 coders. Descriptive statistics were used to summarize implementation adherence ratings by item and session (ie, 21 items × 2 sessions = 42 ratings for each coder). Overall intercoder agreement among the 3 coders was estimated using Fleiss κ [30]. Pairwise intercoder agreement (coder 1 vs coder 2, coder 1 vs AI coder, and coder 2 vs AI coder) was evaluated using Cohen κ with 95% CIs. In addition, percentage agreement between pairs of coders was calculated to facilitate the interpretation of intercoder agreement statistics. Following standard practice, κ values were interpreted using the benchmarks proposed by Landis and Koch [31], specified a priori, in which values of 0.00 to 0.20 indicate slight agreement, 0.21 to 0.40 fair, 0.41 to 0.60 moderate, 0.61 to 0.80 substantial, and 0.81 to 1.00 almost perfect agreement.

### Ethical Considerations

All procedures involving human participants were reviewed and approved by the University of Miami Institutional Review Board (ID: 20250114; approval date: March 7, 2025). The study was conducted in accordance with the ethical standards of the institutional research committee. Informed consent was obtained through 2 separate processes: facilitators who participated in the original IBEM sessions provided consent to share the transcripts of their sessions. Prior to any analysis, all transcripts were deidentified by removing names and other direct identifiers so that messages submitted for coding did not contain personally identifying information. Deidentified facilitator messages were transmitted to OpenAI’s GPT 4.1 through the API solely for classification; under the API terms in effect at the time of the study, submitted data were not used to train or improve OpenAI models.

## Results

### Identification of MI Techniques

Across the 72 facilitator messages, *open questions* represented the most frequently coded MI technique across the 2 human coders consisting of 29% (n=21) and 31% (n=22) of the messages, respectively. *Giving information* was identified in 19% (n=14) of the messages by both coders. *Affirmations* were coded in 18% (n=13) and 15% (n=11) of the messages, and *closed questions* were coded in 17% (n=12) and 18% (n=13) of the messages, respectively. *Reflective* statements occurred less frequently; *simple reflections* were identified in 6% (n=4) and 4% (n=3) of the messages, and *complex reflections* were identified in 6% (n=4) and 4% (n=3) of the messages, respectively. *Confrontation* was identified in one (1%) instance by coder 2 and not by coder 1. Messages *not otherwise categorized* accounted for 18% (n=13) and 21% (n=15) of the messages. The AI-based coder demonstrated a generally similar coding distribution of facilitator messages. *Open questions* were identified in 28% (n=20) of the messages, followed by *closed questions* (n=16, 22%), *affirmations* (n=14, 19%), and *giving information* (n=14, 19%). *Simple reflections* were identified in 3% (n=2) of the messages, and no *complex reflections* were detected. Messages coded as not categorized comprised 17% (n=12) of the total messages.

**Figure 1.**
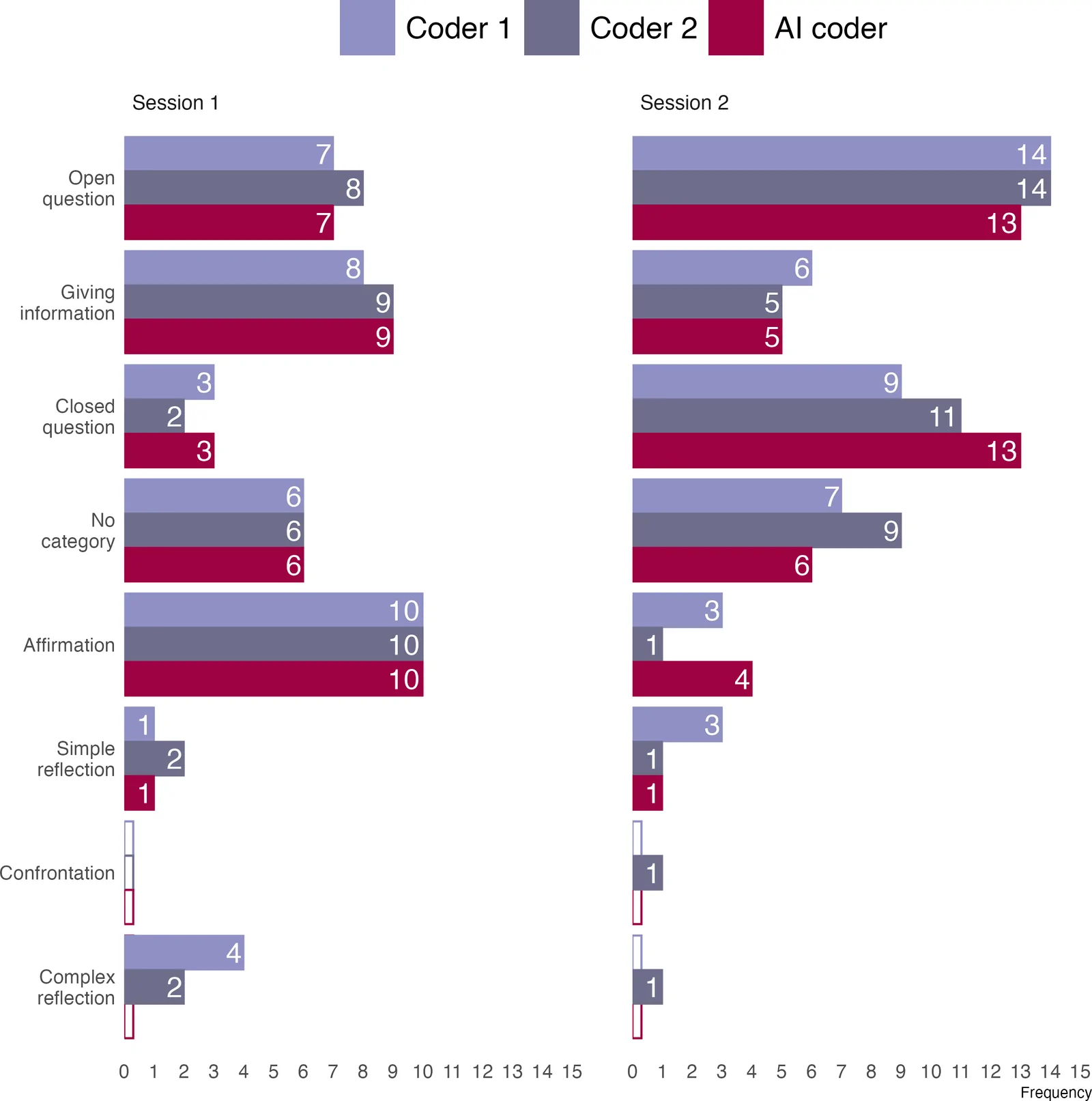
Facilitator message coding frequencies for motivational interviewing (MI) techniques by coder and session.

Cochran *Q* tests revealed no statistically significant differences in the percentages of MI technique use across the 3 coders for any category: *affirmations*, Q(2, n=72)=1.56, *P*=.46; *not categorized*, Q(2, n=72)=1.56, *P*=.46; *open questions*, Q(2, n=72)=0.43, *P*=.81; *closed questions*, Q(2, n=72)=1.73, *P*=.42; *giving information*, Q(2, n=72)=0.00, *P*>.99; *simple reflections*, Q(2, n=72)=1.20, *P*=.55; *confrontation*, Q(2, n=72)=2.00, *P*=.37; and *complex reflections*, Q(2, n=72)=5.20, *P*=.07. The unclear category was not evaluated because it was not endorsed by any coder. The distributions of MI techniques by coder and session are presented in Figure 1.

### Intercoder Agreement

Between the 2 human coders, agreement was high for *giving information* (κ=0.91) and substantial for *affirmations*, *open questions, closed questions*, and messages *not categorized* (κ values ranging from 0.66 to 0.82). Agreement was lower for *complex reflections* (κ=0.55) and lowest for *simple reflections* (κ=0.25). The AI-based coder showed a similar pattern of agreement with both human coders. For the most frequently coded categories (*giving information, affirmations,* and *open questions*), AI-human agreement was substantial to high (κ =0.66-0.91). The main discrepancies involved low-frequency categories: the AI coder did not identify any complex reflections, resulting in κ of 0.00 for both AI-human pairs, and agreement on simple reflections was inconsistent across pairs (κ=0.31 with coder 1 vs κ=0.79 with coder 2). Agreement on closed questions was moderate for the AI-human pairs (κ=0.47 and 0.53), compared with substantial agreement between the two human coders (κ=0.66). Cohen κ coefficients with 95% CIs for all coder pairs are presented in Table 1.

**Table 1.** Cohen κ coefficients and pairwise percentage agreement across pairs of coders^a^.

Category	Coder 1 vs coder 2		Coder 1 vs AI coder		Coder 2 vs AI coder	
	κ (95% CI)	Percentage agreement	κ (95% CI)	Percentage agreement	κ (95% CI)	Percentage agreement
Affirmations	0.70 (0.48 to 0.92)	91.7	0.77 (0.58 to 0.96)	93.1	0.66 (0.43 to 0.89)	90.3
Confrontations	0.00 (0.00 to 0.00)	98.6	—^b^	100	0.00 (0.00 to 0.00)	98.6
Closed questions	0.66 (0.43 to 0.89)	90.3	0.47 (0.22 to 0.72)	83.3	0.53 (0.28 to 0.77)	84.7
Complex reflections	0.55 (0.10 to 1.00)	95.8	0.00 (−0.00 to 0.00)	94.4	0.00 (−0.00 to 0.00)	95.8
Not categorized	0.82 (0.66 to 0.99)	94.4	0.66 (0.43 to 0.89)	90.3	0.68 (0.46 to 0.90)	90.3
Open questions	0.70 (0.52 to 0.88)	87.5	0.69 (0.51 to 0.88)	87.5	0.66 (0.47 to 0.85)	86.1
Giving information	0.91 (0.79 to 1.00)	97.2	0.91 (0.79 to 1.00)	97.2	0.91 (0.79 to 1.00)	97.2
Simple reflections	0.25 (−0.20 to 0.70)	93.1	0.31 (−0.19 to 0.80)	94.4	0.79 (0.40 to 1.00)	98.6

a Interpretive thresholds for κ are as follows: <0.00=poor; 0.00–0.20=slight; 0.21–0.40=fair; 0.41–0.60=moderate; 0.61–0.80=substantial; and 0.81–1.00=almost perfect.

b This category did not occur and κ could not be estimated.

### IBEM Implementation Adherence

**Figure 2.**
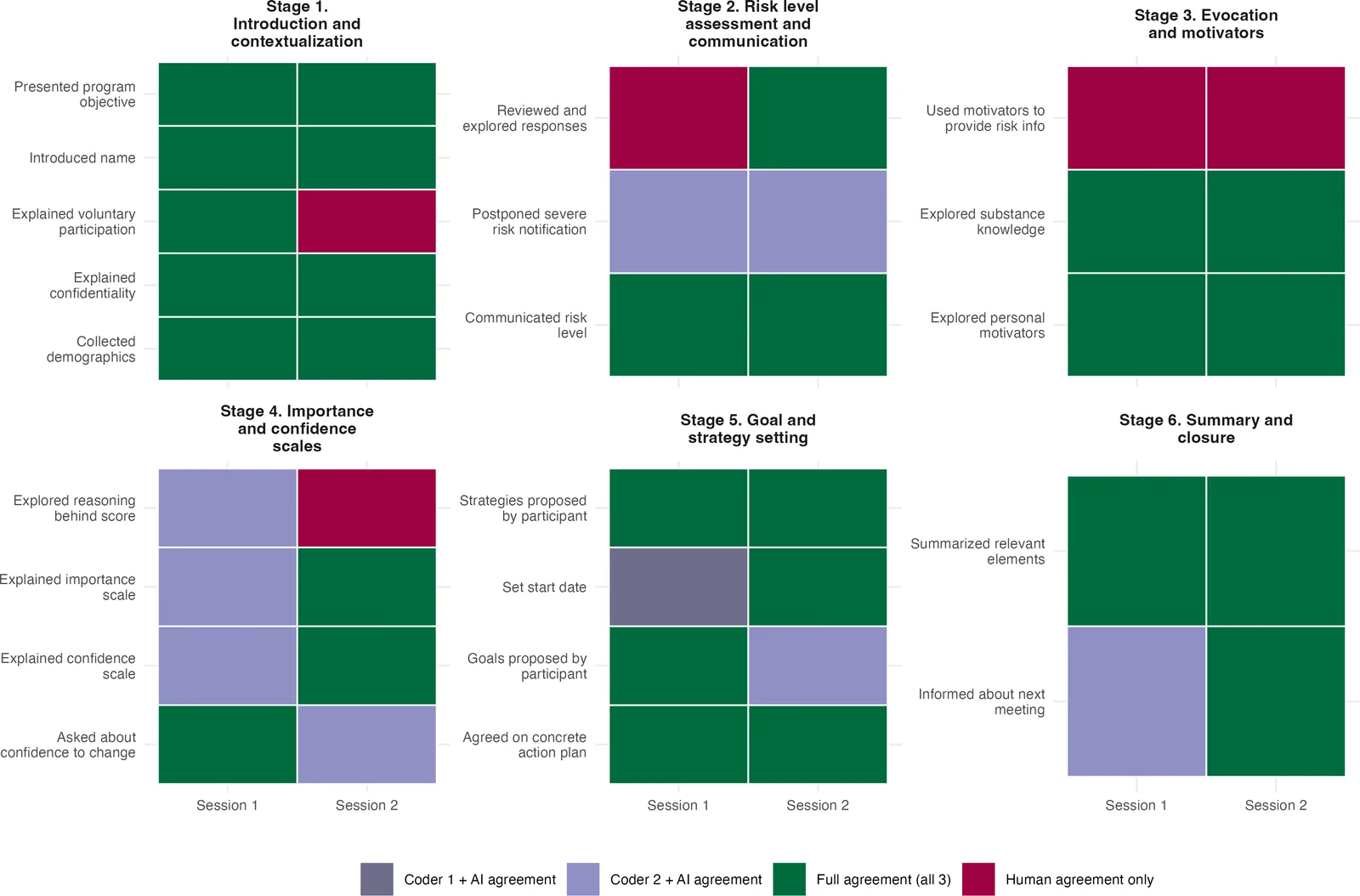
Agreement among coders (human and AI) for Intervención Breve Basada en Entrevista Motivacional (IBEM) implementation adherence items by stage.

Across the 2 IBEM sessions, adherence to implementation stages varied by session but showed substantial overlap among AI and human coders. Using the 21-item checklist, coders 1 and 2 endorsed 12 (57.1%) and 10 (47.6%) items in session 1, respectively, whereas the AI coder endorsed 11 (52.4%). In session 2, endorsement rates were higher for all coders (coder 1: n=16, 76.2%; coder 2: n=17, 81.0%; AI coder: n=20, 95.2%). When checklist ratings were pooled between sessions (42 ratings), full 3-way agreement occurred for 28 (66.7%) of the items. In terms of pairwise percentage agreement, concordance was 85.7% between the AI coder and coder 2, 78.6% between the 2 human coders, and 69.0% between the AI coder and coder 1. Disagreements clustered primarily in stage 2 (Risk Assessment) and stage 4 (Importance and Confidence), whereas stage 1 (Presentation and Contextualization) showed near-uniform agreement across coders. Overall, interrater agreement across the 3 coders, assessed with Fleiss κ, was moderate (κ=0.487, *z*=5.47; *P*<.001). Pairwise Cohen κ indicated moderate agreement between the 2 human coders (κ=0.526, 95% CI 0.256-0.797) and substantial agreement between coder 2 and the AI coder (κ=0.669, 95% CI 0.432-0.907), whereas agreement between coder 1 and the AI coder was fair (κ=0.264, 95% CI 0.043-0.571). Figure 2 presents agreement patterns for each adherence item by the stage of IBEM implementation across the 2 IBEM sessions.

For each of the 21 checklist items in each of the 2 sessions, “agreement” denotes concordance among coders on the same present (1) or absent (0) rating. The figure displays both 3-way agreement (all 3 coders assigning the same rating) and pairwise agreement, organized by the 6 IBEM implementation stages.

## Discussion

### Principal Findings

This study examined the use of an LLM to automate the identification of intervention techniques and implementation adherence coding within the context of a brief MI-based preventive intervention for adolescents. Agreement between the AI-based coder and trained human coders was assessed at 2 levels: individual facilitator messages for MI technique identification and item-level checklists for session-specific implementation adherence. Using the framework of Carroll et al [17], these may be thought of as implementation quality of delivery and adherence, 2 core components of implementation fidelity. Proctor et al [32] position fidelity as a key implementation outcome that mediates the relationship between implementation strategies and intervention effectiveness, underscoring the importance of scalable fidelity measurement. Overall, our findings provide support for the feasibility of AI-assisted coding of MI techniques and implementation adherence, although they reveal specific limitations that require further investigation. These results were derived from a proof-of-concept dataset and were therefore intended to establish initial feasibility rather than to justify large-scale deployment or to guide consequential coding decisions in practice. Accordingly, the estimates reported below should be interpreted with caution.

Consistent with our first hypothesis, the AI-based coder demonstrated moderate-to-substantial agreement with human coders across the most commonly used MI techniques. Agreement was particularly strong for *giving information*, *affirmations*, and *open questions*, techniques characterized by relatively explicit linguistic markers that may be easier for automated detection [10]. Importantly, Cochran *Q* tests revealed no statistically significant differences in the percentages of MI technique use across the 3 coders for any technique category, supporting our hypothesis that the AI system would not exhibit systematic bias in category assignment. These agreement levels are broadly consistent with those reported in prior automated MI coding research. For example, Tanana et al [9] found that both deep-learning models achieved high agreement with human coders for open and closed questions, giving information, and affirmations (all κ>.50), with modestly lower agreement for simple and complex reflections (κ values between 0.30 and 0.50). The present study, using a general-purpose LLM without task-specific training, achieved comparable or higher agreement for these high-frequency categories, suggesting that advances in language modeling may reduce the need for domain-specific model training.

We note, however, that the AI coder’s performance on *reflection* statements was notably weaker than other MI techniques. The AI system failed to identify any *complex reflections*, producing κ values of 0.00 for both human-AI comparisons. *Complex reflections* require inferring implicit meaning, emotional undertones, and rephrasing that extends beyond the literal content of participant statements, processes that demand a level of pragmatic interpretation that current LLMs struggle with [13]. Notably, *complex reflections* were also among the least frequently coded categories by human raters, and agreement between the 2 human coders on this category was only moderate (κ=0.55), suggesting that these distinctions are inherently difficult even for trained experts. For simple reflections, an interesting asymmetry emerged: agreement between the AI and coder 2 was substantial (κ=0.79), whereas agreement with coder 1 was fair (κ=0.31). This variability likely reflects individual differences in how coders operationalize the boundary between *simple reflections* and similar categories such as *closed questions* or paraphrases, a well-documented challenge in MI coding [33], and suggests that coding MI techniques is a difficult task even for human experts. The AI coder’s inability to detect complex reflections echoes findings from earlier NLP-based approaches. Tanana et al [9] similarly reported lower accuracy for reflections compared with questions, and Atkins et al [10] noted that categories requiring pragmatic inference have the greatest challenge for statistical classifiers. The present findings suggest that this limitation persists even with more advanced language models.

The results from the IBEM 21-item implementation adherence checklist were similarly promising. Full 3-way agreement occurred for two-thirds of the checklist items (n=28, 66.7%), and the AI coder achieved the highest pairwise percentage agreement with coder 2 (n=36, 85.7%). Pairwise κ between the AI coder and coder 2 was substantial (κ=0.66), exceeding the moderate agreement observed between the 2 human coders themselves (κ=0.52). These findings suggest that the AI coder was able to interpret session structure and procedural adherence with a degree of reliability comparable to human-to-human agreement. Checklists are commonly used in implementation settings to monitor facilitator compliance with program protocols [34], and the present results indicate that AI-based monitoring could meaningfully support this function and speed up the pace of adherence monitoring.

That said, agreement between the AI coder and Coder 1 was only fair (κ=0.264), indicating unreliable concordance for this pair. This discrepancy, however, should be interpreted in light of the disagreement among the human coders themselves rather than as evidence of a problem unique to the AI coder. The 2 human coders agreed only moderately on adherence (κ=0.52), so there was no single human “gold standard” against which the AI coder could be benchmarked. Critically, the AI coder aligned substantially with coder 2 (κ=0.66) but only fairly with coder 1, and coder 1 also diverged from coder 2 to a comparable degree. Coder 1 was therefore the most distinct rater of the three, and the AI coder’s weaker agreement with that coder largely mirrors a preexisting difference between the 2 humans.

Disagreements clustered in stage 2 (Risk Assessment) and stage 4 (Importance and Confidence), suggesting that adherence checklist items requiring interpretation of facilitator intent, rather than the presence of explicit content, remain challenging for both human and AI coders. It is also worth noting that checklist-based adherence assessment has inherent limitations: sessions are highly sensitive to the context of each interaction. For instance, some adolescents may be more engaged or collaborative, while others may be more reserved, leading facilitators to adapt their delivery accordingly. These contextual variations may produce natural differences in how facilitators approach each stage, which in turn complicates the interpretation of adherence ratings regardless of whether the coder is human or AI.

From an implementation science perspective, the AI coder’s ability to consistently recognize most core MI techniques and procedural steps represents a meaningful advancement for scalable fidelity monitoring. Returning to the conceptualization of implementation fidelity of Carroll et al [17], the AI coder performed well on adherence measurement, where agreement with one human coder exceeded human-to-human agreement, and on identification of MI techniques (a potential indicator of quality of intervention delivery) for categories with explicit linguistic markers. This pattern suggests that LLM-based coding may be most immediately viable for the adherence dimension of fidelity, which relies on detecting the presence or absence of prescribed intervention components, while the quality of delivery dimension of fidelity requires further development for higher-inference techniques such as complex reflections. Manual coding of MI sessions is time-intensive and costly, and its reliance on trained human coders limits the frequency of fidelity monitoring. An AI-assisted approach could reduce human workload, increase the feasibility of conducting routine fidelity checks, and enable more timely feedback to facilitators, particularly in resource-constrained or geographically dispersed implementation settings. In practical terms, these results point toward the need for a human-in-the-loop workflow rather than full automation. In such a model, the AI coder would “precode” facilitator messages and adherence items and flag low-confidence or higher-inference cases, such as complex reflections and the stage 2 and stage 4 items where agreement was the weakest.

Finally, a human-in-the-loop workflow should treat the AI coder as an annotation instrument and assess whether its outputs systematically change the distribution of codes across categories. For example, Xu et al [35] found that an LLM annotator shifted the overall distribution of emotional-tone labels toward the “very negative” category. In our study, the AI coder identified no complex reflections, whereas the human coders identified some examples of this higher-inference technique. Although these counts are too small to establish systematic undercoding, they illustrate why human oversight should examine whether the AI consistently over- or underrepresents specific categories before its codes are used in fidelity monitoring or as training labels.

### Limitations

Several limitations should be considered when interpreting these findings. First, the study analyzed only 2 session transcripts comprising 72 facilitator messages and 42 adherence checklist items, which limits the generalizability of the study findings. With the small number of observations per category, the agreement estimates have limited precision, as reflected in the wide CIs, and the results should be regarded as preliminary evidence. Second, only 2 human coders participated, which restricts the estimation of human-to-human reliability and does not capture the full range of variability that would be observed in a larger coder panel. Because human-to-human reliability was itself moderate, comparisons between the AI coder and each individual coder were sensitive to that coder’s idiosyncratic coding style. A larger and more diverse panel of coders would provide a more stable human reference standard against which to benchmark the AI coder. Third, the study evaluated a single LLM (GPT 4.1) with a single prompt configuration; performance may differ across models, prompt versions, and prompt engineering strategies, and the study results should not be generalized to other LLMs without direct comparison. Fourth, all transcripts were in Spanish from a Colombian intervention context, and it remains unknown whether agreement levels would hold for MI sessions conducted in other languages or cultural settings. Relatedly, the transcripts were drawn from standardized facilitator practice sessions in which the interlocutor was a chatbot simulating adolescent responses rather than a live client; although this design isolated facilitator MI behaviors under controlled conditions, conversational dynamics may differ from naturalistic school-based sessions, and the findings should be replicated with live adolescent interactions before broader use. Finally, the coding framework used in this study focused on behavioral counts of MI techniques and binary adherence checklist items; the study did not evaluate higher-order global competencies such as empathy, which may present additional challenges for automated classification.

Future research should address these limitations by (1) expanding the number and diversity of transcripts to include sessions from multiple intervention sites and facilitators, (2) comparing the performance of multiple LLMs and systematically evaluating the impact of prompt engineering on classification accuracy, (3) examining whether AI-assisted feedback can improve both proximal (eg, facilitator adherence) and distal (eg, program sustainability) implementation outcomes in prospective designs, and (4) extending the coding framework to include global MI competencies (eg, empathy) to test automated coding of complex MI techniques. Additionally, cross-linguistic validation studies are needed to determine whether the present findings generalize to MI sessions conducted in English and other languages. Integrating AI-based coding into real-time supervision workflows represents a particularly promising direction for translating these findings into implementation practice.

## Supplementary material

10.2196/95964 Checklist 1 GRRAS checklist.

10.2196/95964 Checklist 2 TRIPOD-LLM checklist.

## Abbreviations

GRRAS: Guidelines for Reporting Reliability and Agreement Studies
IBEM: Intervención Breve Basada en Entrevista Motivacional [Brief Intervention Based on Motivational Interviewing program]
LLM: large language model
MI: motivational interviewing
MISC: Motivational Interviewing Skill Code
MITI: Motivational Interviewing Treatment Integrity
NLP: natural language processing
OARS: open questions, affirmations, reflections, and summaries
TRIPOD-LLM: Transparent Reporting of a Multivariable Prediction Model for Individual Prognosis or Diagnosis–Large Language Models
