# Supplementary material for: Automating Motivational Interviewing Coding in Adolescent Substance Use Prevention: Human-AI Agreement Study
Source: JMIR AI. 2026 Aug 25;5:e95964. doi: 10.2196/95964 (PMC13505344; doi:10.2196/95964)
Supplement: Supplementary file 2 [file ai-v5-e95964-s001.docx]

**GRRAS Checklist.**

**Guidelines for Reporting Reliability and Agreement Studies**

Based on Table I in: Kottner J, Audigé L, Brorson S, Donner A, Gajewski BJ, Hróbjartsson A, Roberts C, Shoukri M, Streiner DL. Guidelines for reporting reliability and agreement studies (GRRAS) were proposed. J Clin Epidemiol. 2011;64(1):96–106.

| Section | Item # | Checklist item | Reported in |
| --- | --- | --- | --- |
| Title/Abstract | 1 | Identify in title or abstract that interrater/intrarater reliability or agreement was investigated. | Title (“A Human–AI Agreement Study”) and Abstract (intercoder agreement assessed using Cohen’s κ, Fleiss’ κ, and Cochran’s Q). |
| Introduction | 2 | Name and describe the diagnostic or measurement device of interest explicitly. | Coding Framework (MI technique categories and 21-item IBEM adherence checklist). |
|  | 3 | Specify the subject population of interest. | Intervention Context (adolescents in a school-based substance use preventive intervention in Colombia). |
|  | 4 | Specify the rater population of interest (if applicable). | Participants (certified IBEM facilitators with psychology degrees, MI training, and 5+ years of experience) and AI-based Coding (OpenAI gpt-4.1). |
|  | 5 | Describe what is already known about reliability and agreement and provide a rationale for the study (if applicable). | Introduction (reviews prior NLP-based MI coding efforts, identifies gap for LLMs in non-English preventive interventions). |
| Methods | 6 | Explain how the sample size was chosen. State the determined number of raters, subjects/objects, and replicate observations. | IBEM Transcripts (2 transcripts selected from a pool of 10 sessions; 72 facilitator messages; 3 coders; 21-item checklist × 2 sessions = 42 adherence ratings per coder). |
|  | 7 | Describe the sampling method. | IBEM Transcripts (purposive selection based on three criteria: typical delivery, average session length, coders not assigned to own sessions). |
|  | 8 | Describe the measurement/rating process (e.g., time interval between repeated measurements, availability of clinical information, blinding). | Procedures (structured virtual training; independent coding of two transcripts; individual meetings with research team after first transcript; minor refinements before second transcript). |
|  | 9 | State whether measurements/ratings were conducted independently. | Study Design (“coded independently”) and Procedures (“Coders were blinded to one another’s ratings and to the AI-generated codes throughout the process”). |
|  | 10 | Describe the statistical analysis. | Statistical Analysis (Cohen’s κ for pairwise agreement by technique; Cochran’s Q for systematic differences; Fleiss’ κ for overall three-way adherence agreement; 95% CIs; percentage agreement). |
| Results | 11 | State the actual number of raters and subjects/objects which were included and the number of replicate observations which were conducted. | Results MI Techniques (3 coders, 72 facilitator messages across 2 sessions) and IBEM Implementation Adherence (42 adherence ratings per coder). |
|  | 12 | Describe the sample characteristics of raters and subjects (e.g., training, experience). | Participants (Colombian professionals, psychology degrees, postgraduate training, IBEM certification requiring 32-hour training, 5+ years implementation experience) and AI-based Coding (gpt-4.1 with structured prompt derived from coding manual). |
|  | 13 | Report estimates of reliability and agreement including measures of statistical uncertainty. | Intercoder Agreement (Cohen’s κ with 95% CIs in Table 1); IBEM Implementation Adherence (Fleiss’ κ = .487, z = 5.47, p < .001; pairwise Cohen’s κ with 95% CIs; percentage agreement); Cochran’s Q tests with p-values. |
| Discussion | 14 | Discuss the practical relevance of results. | Discussion (feasibility of AI-assisted fidelity coding; human-AI collaborative model; implications for scalable implementation monitoring in resource-constrained settings). |
| Auxiliary material | 15 | Provide detailed results if possible (e.g., online). | Table 1 (full κ coefficients with CIs); Figure 1 (MI technique frequencies by coder and session); Figure 2 (adherence agreement by stage); Availability of Data and Materials (datasets available from corresponding author on reasonable request). |
